# Book Reviews

**Published:** 1895-08

**Authors:** 


### Book Reviews, Etc.

Twentieth Century Practice of Medicine, Vol. III. William Wood & Co., New York.

The third volume of this most elaborate work on practice of medicine contains descriptions of occupation diseases, drug habits and poisons. The division of the work devoted to drug habits covers one hundred and thirty-seven pages, and is written by Dr. Norman Kerr, of London, and is chiefly devoted to a discussion of pathological effects of alcoholism, morphinism, and chloralism, with an extended study of inebriety.

The subject of "Shock and Collapse," is ably managed by Dr. F. Shrady.

The article on "Seasickness—Naupathia," by Dr. A. L. Gihon, of the U. S. Navy, contains a full account of this very little understood complaint. Dr. Gihon quotes several fatal cases of the *mal de mer*, showing that it is not such a trivial complaint as is generally thought. He divides the cases into three classes: the moderately, considerably, or severely affected, and discusses the symptoms and treatment of each class.

The section on diseases of occupation is very exhaustive, and is written by the well-known neurologist, Dr. James Hendrie Lloyd, of Philadelphia.

In that on "Poisoning" by Beaumont Small, of Ottawa, an accurate cut is given of the poisonous plants, with their seed or fruit, considered in the text.

The article on "Osteomalacia," is by Councilman, of Harvard, and that on "Mountain Sickness," by Georg von Liebig, of the University of Munich.

"Hæmidrosis and Bromidrosis," Isadore Dyer, M. D., New Orleans.

"A Curious Anomaly of the Female Genitalia, etc.," by W. A. H. Coop, M. D., Lawrenceburg, Tenn.

"Bicycling for Women, from a Standpoint of the Gynæcologist," by R. L. Dickinson, M. D., Brooklyn, N. Y.

